# Editorial: Systems biology strategies in parasitic diseases

**DOI:** 10.3389/fcimb.2023.1192732

**Published:** 2023-04-12

**Authors:** Laurence A. Marchat, Juan David Ospina-Villa, María Esther Ramírez-Moreno

**Affiliations:** ^1^ Escuela Nacional de Medicina y Homeopatía, Instituto Politécnico Nacional, Mexico, Mexico; ^2^ Instituto Colombiano de Medicina Tropical, Universidad CES, Antioquia, Colombia

**Keywords:** bioinformatics, diagnosis, eukaryotic parasites, host-pathogen interactions, omics data, parasite biology, system biology, treatment

At the beginning of the XXI century, as the COVID-19 pandemic recently remembered us, infectious diseases still threaten human health in both developing and developed countries. Notably, the World Health Organization reported that they are directly responsible for 15% of all deaths worldwide, making them a challenging area of investigation. Beside bacteria and virus, eukaryotic parasites produced a large range of symptoms that lead to disfiguring, debilitating and lethal diseases besides the effectiveness of the currently available drugs. Additionally, their side effects, the existence of drug resistance and the absence of vaccines strongly justify the need for new methods for controlling these parasites. In this context, genome sequence analyses, multi-omics strategies and the computational integration of multi-omics data mainly based on transcriptomics, and proteomics, have provided key information about the biological molecular functions and networks supporting these living systems, that can be applied to identify key proteins and events with potential for the development of more effective prevention, prophylaxis, diagnosis, and treatment methods.

Among protozoan parasites*, Entamoeba histolytica*, the causative agent of human amoebiasis, causes endemic infections in developing countries and population living in poor sanitary conditions. Developed countries are also concerned since it affects travelers returning from endemic areas, and immunocompromised individuals. The main characteristics of amoebiasis include colitis, severe diarrhea, dysentery, and liver abscesses, that are responsible for 40,000–100,000 deaths annually. *E. histolytica* is considered as the fourth leading cause of death due to a parasite infection, and therefore requires additional molecular studies for the development of enhanced diagnostic capacity and disease surveillance.

One of the virulence hallmarks of *E. histolytica* is phagocytosis, which involves the participation of the Endosomal Sorting Complexes Required for Transport (ESCRT) machinery for endocytosis, endosome and multivesicular bodies (MVBs) formation, as well as cargo delivery into lysosomes. Notably, ESCRT-I and ESCRT-III components, other proteins such as EhADH adhesin, EhRabs, and actin, and the lysobisphosphatidic acid (LBPA) contribute to membrane remodeling. Using a proteomic approach, Banuelos et al. showed that LBPA is associated to a set of proteins related to membrane trafficking, cellular transport, cytoskeleton dynamics, and transcriptional and translational functions, that are secreted.

The proteomic approach can also give new insights about the molecular mechanisms of new anti-parasite drugs. Thus, Avila-Bonilla et al. chose this strategy to elucidate the effect of two quinoxaline derivatives (QdNO) named T-001 and T-017 on *E. histolytica* trophozoites. Changes in the abundance of proteins mainly related to redox homeostasis, cytoskeleton and intracellular traffic, suggested that QdNO affect essential functions related to the actin cytoskeleton, which ultimately has a negative impact on *E. histolytica* virulence and survival.

Amebic liver abscess (ALA) is the most common and severe extraintestinal manifestation of amebiasis. In agreement with the relevance of anti-amoebic oxidative intermediates for liver tissue damage, Cruz-Baquero et al. showed the effectiveness of the antioxidant activity of ascorbic acid (ASC) to reduce liver damage and parasites in an experimental model of ALA in hamsters. They also revealed the effect of ASC on the myeloperoxidase (MPO) enzyme amount and activity to restore the biological liver system.

Finally, the identification of circRNA-producing genes and their association with encystment, sulfur metabolism and virulence allowed González-Blanco et al. to propose that circRNAs might have acquired new roles in the regulation of various cellular functions during the evolution of *Entamoeba* species.

Helminths are another group of parasites of importance for human health. Transmission occurs when parasite eggs present in human feces contaminate the soil, food, or water in areas with deficient sanitation systems. The soil-transmitted helminths (STH) cause the most common infections worldwide with an estimated 1.5 billion infected people. In addition to health and hygiene education, periodic treatments with routine anti-helminth therapy, mainly albendazole and mebendazole, are necessary to reduce the intensity of infection, and therefore morbidity.

The cestode parasite *Taenia crassiceps* is commonly used as an animal model for studying the human cysticercosis caused by the zoonotic tapeworm *Taenia solium* in Latin America, South and South-East Asia and sub-Saharan Africa. From the characterization of the genome sequences and the analysis of gene expression, Bobes et al. achieved to propose a new hypothesis to explain the absence of scolex in the ORF strain of *T. crassiceps*. They also highlighted the potential of comparative genomics for the design of new methods for parasite control.

The STH *Ascaris* and *Trichuris* represent relevant parasite nematodes in low- and middle-income regions, affecting both human and animal well-being. They can survive and persist in the human host for long period of time, which indicate the existence of complex relationships between both biological systems. As Castañeda et al. show in their interesting review, studies based on metagenomics, metataxonomics and metabolomics strategies contributed to the understanding of the effect of parasite infections on the composition and structure of the host microbiota, which in turn has an impact on pathogen biology, infection dynamics and pathophysiology.

Although the studies contained in this Research Topic only focused on a small number of the eukaryotic parasites that affect human health, their results provide interesting data and confirm the potential of omics and bioinformatics strategies for a better understanding and control of these pathogens.

## Author contributions

All authors listed have made a substantial, direct, and intellectual contribution to the work and approved it for publication.

